# Dramatic Remodeling of Hypoplastic Pulmonary Arteries by Pulmonary Valve Opening, Right Ventricular Outflow Stenting, and Aortopulmonary Collateral Lung Territory Incorporation

**DOI:** 10.1016/j.jscai.2026.104270

**Published:** 2026-03-12

**Authors:** Marjan Hesari, Derek Van, Srujan Ganta, Justin Ryan, Jessica Haley, Jose Silva Sepulveda, Rohit Rao, Brent Gordon, John Nigro, Howaida G. El-Said

**Affiliations:** a Department of Pediatrics, University of California, San Diego, La Jolla, California; b Department of Human Biology, University of California, San Diego, La Jolla, California; c Department of Surgery, University of California, San Diego, La Jolla, California; d Helen and Will Webster Foundation 3D Innovations Lab, Rady Children's Hospital, San Diego, California; e Department of Neurological Surgery, UC San Diego Health, La Jolla, California

**Keywords:** pulmonary artery, pulmonary atresia, pulmonary valve, right ventricular outflow tract stenting, tetralogy of Fallot

## Abstract

**Background:**

Pulmonary atresia with severely hypoplastic pulmonary arteries (PAHP), with or without major aortopulmonary collateral arteries (MAPCA), is a significant clinical conundrum. This case series aims to present a strategy being evaluated for patients with PAHP, highlighting the effectiveness of pulmonary valve opening, right ventricular outflow tract (RVOT) stenting, and staged serial interventions in promoting pulmonary artery (PA) growth and improving PA architecture. This study also describes a technique of dilation of the junction between the distal PA and the collaterals to incorporate them into the “true PA territory,” potentially negating the need for unifocalization.

**Methods:**

A retrospective review was performed of 4 patients with PA/HP/MAPCA treated with pulmonary valve opening, RVOT stenting, and PA balloon rehabilitation. Procedural techniques, outcomes, and PA growth are described.

**Results:**

The procedure was successful in 3 of the 4 patients, all without major complications. In these patients, significant PA growth was achieved, with the Nakata index increasing from a baseline median of 10.8 to as high as 483.6. Dilation of a connection between a MAPCA and the true PA was performed in 1 patient, allowing recruitment of additional lung territory. All 3 subsequently completed septation, with 2 requiring unifocalization of only a single collateral vessel. In the fourth patient, unfavorable RVOT–main PA malalignment prevented successful wire crossing; thus, right ventricular perforation/stenting was not performed.

**Conclusions:**

Pulmonary valve opening and RVOT stenting with PA rehabilitation, including dilation of the connections of the MAPCA to the true PA, is a promising strategy to promote PA growth in PAHP with MAPCA, facilitating progression to surgical repair while minimizing the need for unifocalization.

## Introduction

Pulmonary atresia and severely hypoplastic pulmonary arteries (PAHP), with or without major aortopulmonary collateral arteries (MAPCA), are significant clinical conundrums.^1^^,^^2^ The clinical presentation varies depending on the source and amount of pulmonary blood flow, with poor survival outcomes in the absence of timely intervention.^3^ The pulmonary artery (PA) size is a significant factor in determining long-term outcomes.^4^^,^^5^ Several approaches have been utilized to achieve PA growth, including modified Blalock-Thomas-Taussig shunt and surgical right ventricular outflow tract (RVOT) patch augmentation.^6^^,^^7^ Recent studies, including a large cohort by Petrucci et al,^8^ highlighted the nonnegligible mortality (7.2%) and morbidity (13.1%) associated with neonatal surgical shunts. Although surgical repair remains the ultimate therapeutic goal, catheter-based interventions have emerged as valuable adjuncts or alternatives, particularly in high-risk neonates.^9^ Percutaneous approaches, such as RVOT stenting, offer a promising means to establish reliable pulmonary blood flow in tetralogy of Fallot,^10^ and pulmonary valve perforation has been shown to promote right ventricular (RV) growth in pulmonary atresia with intact ventricular septum (PA/IVS).^11^^,^^12^ Our group and others previously reported the technical feasibility of radiofrequency perforation of the pulmonary valve in the setting of PA/ventricular septal defect (VSD).^13^^,^^14^

This case series aims to present a strategy being evaluated for patients with PAHP, highlighting the effectiveness of pulmonary valve opening, RVOT stenting, and staged serial interventions in promoting PA growth and improving PA architecture. This study also describes a technique of dilation of the junction between the distal PA and the collaterals to incorporate them into the “true PA territory,” potentially negating the need for unifocalization.

## Materials and methods

This is a retrospective review of 4 patients presenting with PAHP with MAPCA who were managed in Rady Children's Hospital with pulmonary valve opening and RVOT stenting between 2018 and 2025.

Patients considered for this approach are those with a definable main pulmonary artery (MPA) target vessel (even if tiny) and an RVOT amenable to wire crossing. Preprocedural computed tomography (CT) angiography with 3D segmentation (Mimics Medical, version 27) was performed in all cases to assess the proximity and orientation between the RVOT and the true MPA, with final feasibility determined at the time of catheterization by angiography.

Patient characteristics, including diagnosis, arch sidedness, comorbidities, age, and weight at intervention, were collected. Procedural data included details of pulmonary valve opening, RVOT stenting, and selective balloon dilation of segmental branches and the junction between PA branches and collateral arteries. All catheter-based and surgical interventions, along with the timing and type of each procedure, were recorded.

**Figure 1:**
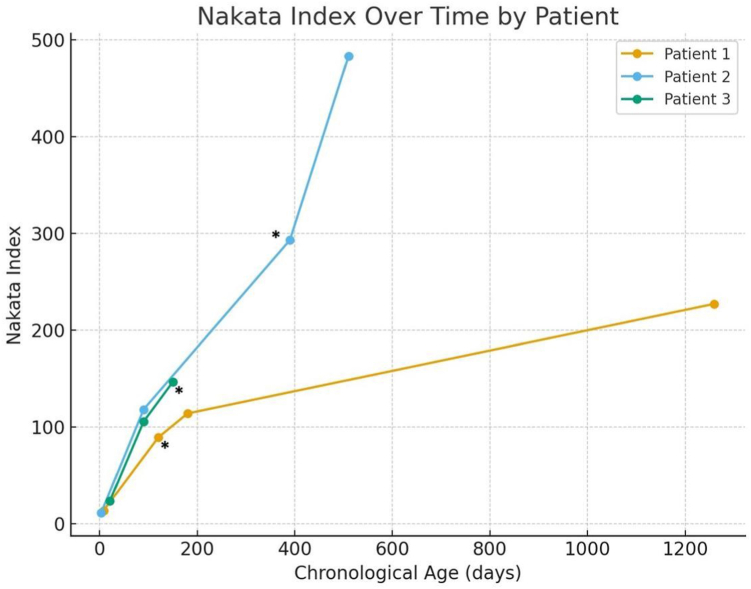
**Nakata index measurements at different key time points.** The figure demonstrates a progressive increase in pulmonary artery size over time. The asterisk represents the last catheterization prior to surgical repair. Case 1 had surgery at 6 months of age. Case 2 had surgery at 13 months of age.

Serial imaging, including CT scans and cardiac catheterization, was used to assess PA growth over time. The dimensions of MPA, right pulmonary artery (RPA), and left pulmonary artery (LPA) were measured at baseline (birth), before the surgical intervention, and at the latest follow-up. The Nakata index was calculated to quantify PA growth^15^ (Figure 1).

## Case 1

A female infant was diagnosed prenatally with PA/IVS, hypoplastic RV with a TV z-score of –3, severely hypoplastic PA, tiny MAPCA, and no ductus arteriosus, a highly unusual and severe combination of single-ventricle physiology and severely underdeveloped pulmonary vasculature. Given the gravity of this condition, she was turned down for intervention by multiple centers.

**Figure 2:**
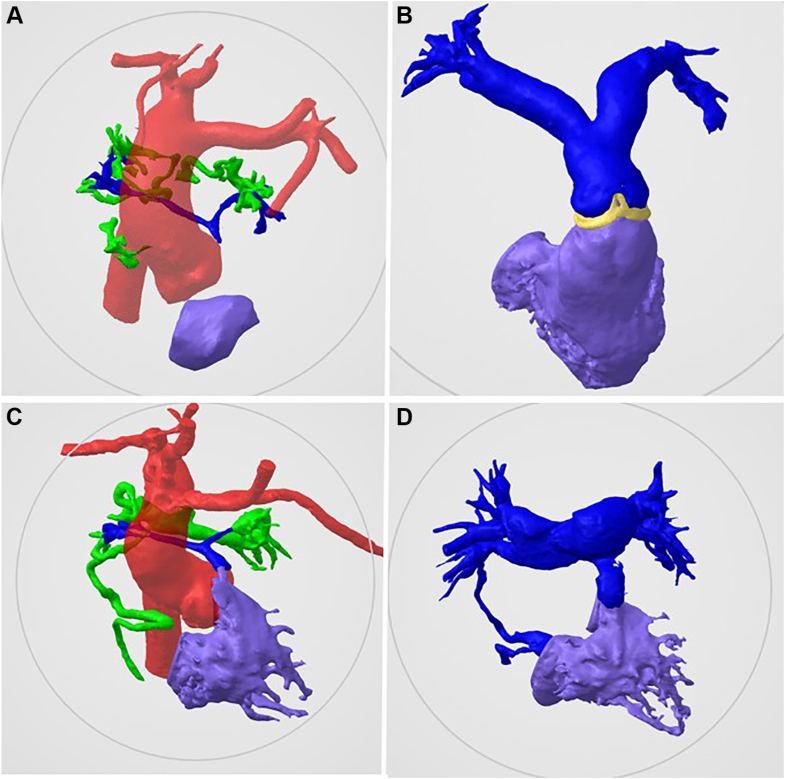
**Three-dimensional reconstruction from computed tomography scan demonstrates interval growth of the pulmonary artery (PA) at birth and at the latest follow-up.** (**A**, **B**) Case 1 with pulmonary atresia with intact ventricular septum, a hypoplastic right ventricle, severely hypoplastic native PA, and tiny major aortopulmonary collateral arteries. (**C**, **D**) Case 2, a patient with pulmonary atresia with ventricular septal defect and major aortopulmonary collateral arteries. Color coding: RV (purple), PA (blue), aortopulmonary collaterals (green), and aorta (red).

She was born at 39 weeks’ gestation, with an initial oxygen saturation of 77%. A CT scan with 3D segmentation was performed and utilized to plan the procedure. The CT scan revealed tiny native PA branches with a Nakata index of 13.38, filling from very small MAPCA arising from the descending thoracic aorta (Figure 2A, B).

At 8 days of life, she underwent radiofrequency perforation of the pulmonary valve followed by stenting of the RVOT using a 4 mm × 12 mm Integrity stent (Medtronic). The distance between the RVOT stump and the MPA was 0.2 mm. A diameter that was a few millimeters larger than the MPA was chosen, and the stent was extended from the mid-MPA into the RVOT to cover the atretic valve without impinging on the branch PA, ensuring that RVOT muscle bundles were crossed.

**Figure 3:**
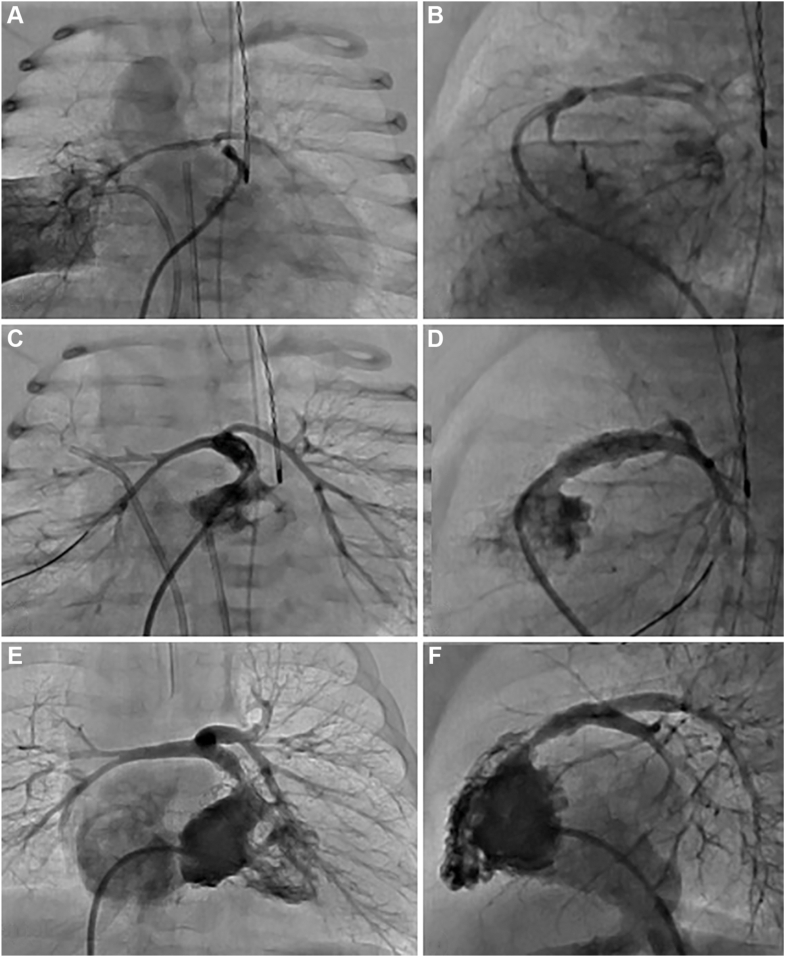
**Case 1.** Serial angiograms in anteroposterior (**A**, **C**, **E**) and lateral (**B**, **D**, **F**) projections. (**A**, **B**) Initial catheterization on day of life 8 prior to pulmonary valve opening, showing simultaneous angiography of the diminutive right ventricular outflow tract via a prograde approach with an angled Glide catheter from the femoral vein, and retrograde filling of hypoplastic branch pulmonary artery (PA) via a wedge angiogram in the right lower pulmonary vein. (**C**, **D**) Postpulmonary valve perforation and right ventricular outflow tract stent placement, revealing improved antegrade flow and enhanced filling of the hypoplastic branch PA. (**E**, **F**) Angiogram at 4 months of age demonstrating interval growth of the branch PA and focal stenosis just proximal to the initial stent, which was successfully restented.

**Figure 4:**
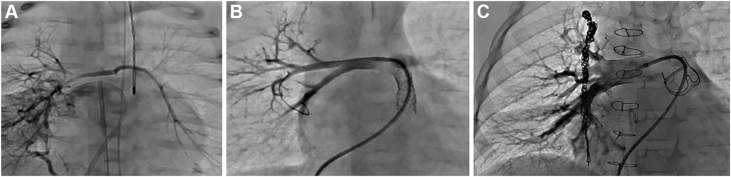
**Case 1.** Angiograms show dramatic growth of the right pulmonary artery (PA) over time. (**A**) Shortly after birth, the right PA is filled via a retrograde angiogram in the right lower pulmonary vein, demonstrating severely hypoplastic branch PA that are markedly underdeveloped with incomplete arborization of the pulmonary arterial tree. (**B**) At 4 months of age prior to surgery, (**C**) at 3 years of age following surgical reconstruction and placement of a bioprosthetic pulmonary valve.

At the time of the procedure, her weight was 3.8 kg. The procedure was complicated by a small pericardial effusion that was drained. Her oxygen saturations at discharge ranged from the high 70s to the low 80s (Figure 3A-D, Figure 4A).

At 4 months of age, she underwent restenting of the RVOT for new obstruction proximal to the original stent, using a 5 mm × 15 mm Resolute Onyx stent (Medtronic) and a 6 mm × 16 mm Formula 418 stent (Cook Medical), along with balloon dilation of both upper and lower branches of the RPA. Over the next several months, significant growth of the branch PA was observed, although her right ventricle remained heavily muscle-bound (Figure 3E, F; Figure 4B).

At 6 months of age, her Nakata index was 88.97 when she underwent surgical removal of the RVOT stent, transannular patch placement, and resection of RV muscle bundles. In this procedure, her ASD was left open, allowing some right-to-left shunting. Her postoperative course was complicated by diaphragmatic paralysis, which was managed conservatively without the need for surgical diaphragm plication.

At 13 and 24 months of age, she underwent 2 additional catheterization procedures for balloon dilation of the right upper, right lower, and left lower PA branches, facilitating further PA growth with a Nakata index of 145.

At 3 years of age, because of persistent cyanosis, she underwent surgical placement of the pulmonary valve with a 19 mm bioprosthetic Inspiris Resilia valve (Edwards Lifesciences), along with branch PA plasty and atrial septal defect closure (Figure 4C).

A few months later, a postoperative cardiac catheterization showed: RV pressure: 29/12 mm Hg (systemic pressure: 90/46 mm Hg), right atrial mean pressure: 9 mm Hg, mean PA pressure: 20 mm Hg, cardiac index: 3.23 L/min/m^2^, and pulmonary vascular resistance: 3.4 Wood units × m^2^. The Nakata index had significantly increased to 227, and her oxygen saturation was 96%.

Across the staged interventions, RV pressures progressively improved as the distal PA obstruction was addressed. After the initial RVOT stent placement, the RV pressure remained suprasystemic (67/16 mm Hg with systemic pressure 50/35 mm Hg), reflecting the persistently severe distal and branch PA hypoplasia. During the second catheterization, additional balloon dilation of the RVOT stent and branch PA decreased the RV systolic pressure to 50 mm Hg with a systemic systolic pressure of 70 mm Hg. Following surgical RVOT resection at the third intervention, RV pressure further declined to half systemic (45/6 mm Hg with systemic pressure 85 mm Hg). At the most recent catheterization, RV pressures reached near-normal levels at 29/12 mm Hg with systemic pressure at 90 mm Hg (Supplemental Video 1).

## Case 2

A male infant, born at 39 weeks via emergency cesarean section because of cord prolapse, was prenatally diagnosed with pulmonary atresia with ventricular septal defect (PA/VSD)/MAPCA. Initial oxygen saturation was 88%.

A CT scan revealed that the pulmonary blood flow was supplied by a large collateral vessel from the descending thoracic aorta, bifurcating into right and left branches. Tiny, small native PA were seen, with a Nakata index of 10.84 (Figure 2C, D).

**Figure 5:**
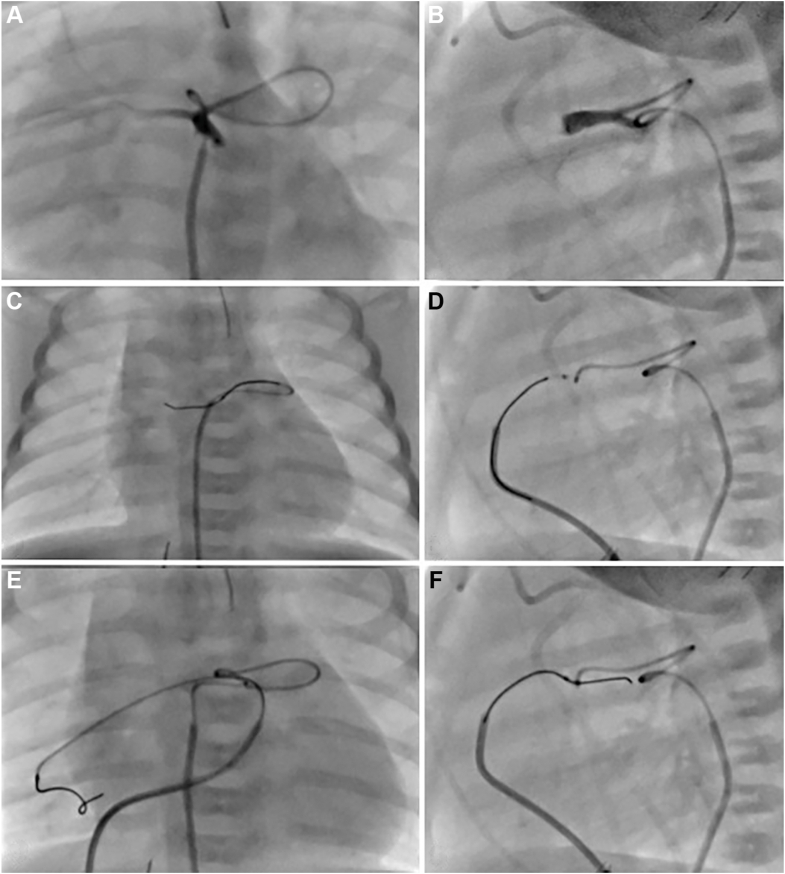
**Case 2.** Serial angiograms in anteroposterior (**A**, **C**, **E**) and lateral (**B**, **D**, **F**) projections during the initial catheterization at 3 days of age, with a Nakata index of 10.84. (**A**, **B**) Angiograms demonstrate severely hypoplastic branch pulmonary artery (PA) with markedly reduced arborization, opacified via a microcatheter positioned through a major aortopulmonary collateral artery arising from the descending aorta. (**C**, **D**) Simultaneous angiography showing 2 microcatheters—one within a major aortopulmonary collateral artery and the other positioned in the right ventricular outflow tract (RVOT)—illustrating the close proximity of the native PA to the RVOT. (**E**, **F**) A BMW wire is shown traversing the pulmonary valve from the RVOT and being advanced into the right lower PA. The wire was intentionally twisted and “locked” distally to anchor and facilitate subsequent balloon angioplasty and stent placement.

**Figure 6:**
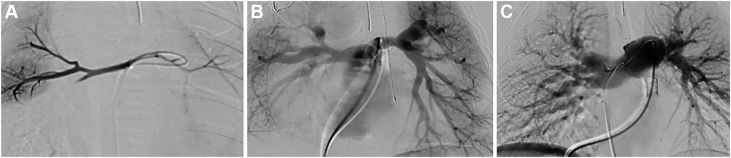
**Case 2.** Serial angiograms illustrate the progression of pulmonary artery (PA) growth in this patient. (**A**) Initial angiogram at 3 days of age demonstrates severely hypoplastic native PA (Nakata index: 10.84), with retrograde filling via a major aortopulmonary collateral artery. (**B**) Angiogram at 12 months of age showing substantial interval growth of the PA (Nakata index: 118.29), with marked improvement in left PA arborization following successful recruitment of the major aortopulmonary collateral artery into the true PA circulation through balloon dilation of the connecting channel. (**C**) Postoperative angiogram following surgical pulmonary arterioplasty, demonstrating well-developed branch PA and a significantly increased Nakata index of 483.6.

At 3 days of age, the patient (weight 3.09 kg) was taken to the catheterization lab, where angiography through an AP collateral revealed a small connection to the native severely hypoplastic PA. The true PA were accessed via the collateral using a Renegade microcatheter (Boston Scientific Corporation) and a BMW wire (Abbott). Angiography of the true PA demonstrated a small MPA. The distance between the RVOT stump and the MPA was 0.8 mm. An angled Glide catheter (Terumo) was placed in the RVOT, revealing a blind pouch. Simultaneous angiographic injections in the MPA and RVOT were performed, and a BMW, through a coaxial system that included an angled glide catheter and Renegade microcatheter, was able to traverse the atretic pulmonary valve. The BMW wire and Renegade were advanced into the distal right lower pulmonary artery (RLPA) branch, and the BMW wire was twisted/locked in position to provide a rail for support.^16^ This stabilization allowed us to advance a 3 mm × 2 cm coronary balloon over the wire to dilate the atretic pulmonary valve (without the need for a long sheath), establishing continuity between the RVOT and PA. Following this, a 4 mm × 12 mm Onyx Frontier stent was deployed across the RVOT. An angiogram was performed through the microcatheter positioned in the MPA (accessed via the collateral) to confirm accurate stent positioning before inflation. Once confirmed, the stent was successfully expanded (Figure 5, Figure 6A).

**Figure 7:**
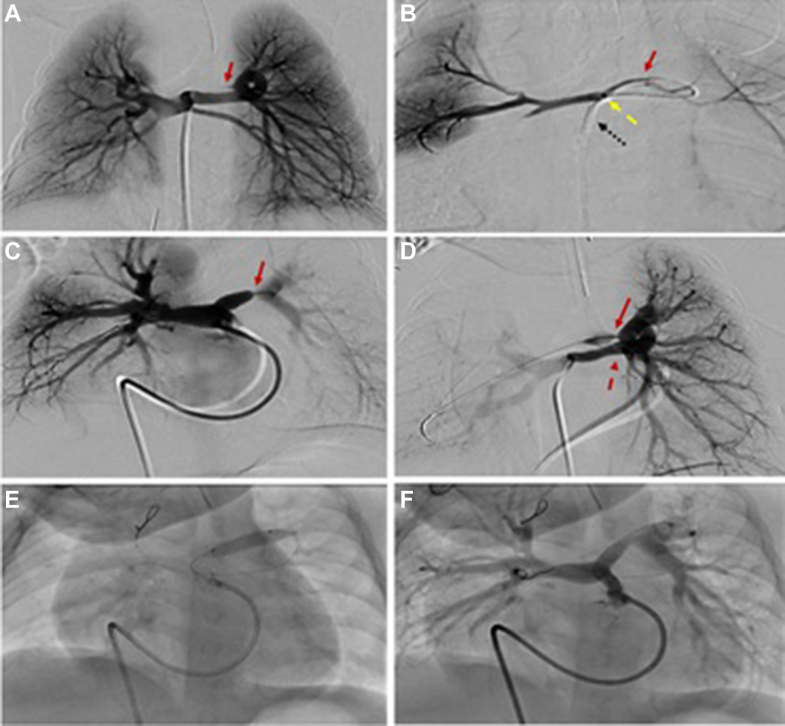
**Case 2.** (**A**) Angiogram demonstrates a large major aortopulmonary collateral artery (MAPCA) supplying both the right and left lungs (solid red line). (**B**) Selective angiogram in the true pulmonary artery (PA). This demonstrates a microcatheter advanced through a large MAPCA into the native, severely hypoplastic PA, accessed retrogradely. The black dotted line indicates an angled Glide catheter positioned at the origin of the MAPCA. The dashed yellow line represents the microcatheter that passed through the Glide catheter and the MAPCA into the connecting channel between the MAPCA and the true PA. The solid red line highlights this critical connection between the MAPCA and the native PA. An angiographic sequence demonstrates successful opening of the connection between the MAPCA and the native PA, with notable interval improvement in the right pulmonary artery (RPA) at 3 months compared to (**A**, **B**) to (**C**, **D**). (**C**, **D**) The solid red arrow highlights a tiny connection between the true left pulmonary artery (LPA) and the MAPCA; the dotted red arrow denotes the course of the MAPCA. (**E**) A guide catheter is positioned in the right ventricular outflow tract, with a wire advanced from the true LPA through the small connection into the MAPCA and further into a right upper lobe branch PA. The wire is twisted/locked distally to anchor balloon advancement. The balloon is shown inflated across the connection between the true LPA and the MAPCA. (**F**) Post–balloon dilation image demonstrates marked improvement in the caliber of the connection. Note the increased diameter of the connection in panel (**F**) compared to the narrow connection in panel (**C**), both marked with red arrows.

At 3 months of age, the patient returned to the catheterization lab for balloon dilation of both the right upper and RLPA branches. During this procedure, a small connection was identified between the distal LPA and a branch of the left-sided MAPCA. Serial balloon dilation of this connection (using 2 mm, then 4 mm, then 5 mm NC emerge balloons [Boston Scientific Corporation]) successfully opened and recruited the larger MAPCA and provided continuity with the LPA (Figure 7). During the same catheterization, a second stent (5 mm × 16 mm Megatron stent [Boston Scientific Corporation]) was placed in the RVOT because of concern about stenosis in the RVOT proximal to the previously placed stent.

At 12 months of age, the Nakata index was 118.29, and the patient underwent surgical unifocalization of only 1 collateral vessel that did not have dual supply, along with PA patch plasty and augmentation of both proximal right and left PA. The RVOT stent was left in place, and the VSD was left open (Figure 6B, Figure 7).

At 13 months of age, the patient returned to the catheterization lab for closure of the remaining connection with the MAPCA. There was a significant improvement in the caliber of the branch PA with a Nakata index of 293.

At 14 months of age, the patient underwent complete intracardiac repair with RVOT patch and VSD closure.

At 17 months of age, he underwent cardiac catheterization to assess hemodynamics and close a residual MAPCA. Baseline hemodynamics showed a cardiac index of 5 L/min/m^2^, RV pressure at 46% of systemic, mean PA pressure of 21 mm Hg, and a transpulmonary gradient of 10 mm Hg. There was an 8 mm Hg gradient from the RPA to the RLPA, with no gradients in any of the other branches. On 100% FiO_2_ and 40 ppm inhaled nitric oxide, mean PA pressure improved to 16 mm Hg and transpulmonary gradient to 5 mm Hg. Balloon angioplasty of the proximal RLPA using 5 mm and 7 mm Powerflex balloons (Cordis) was performed with angiographic improvement. The remaining MAPCA was occluded with a 6.5 mm Siege plug (Merit Medical). The Nakata index was 483.60 mm^2^/m^2^ (Figure 6C, Supplemental Video 2).

## Case 3

**Figure 8:**
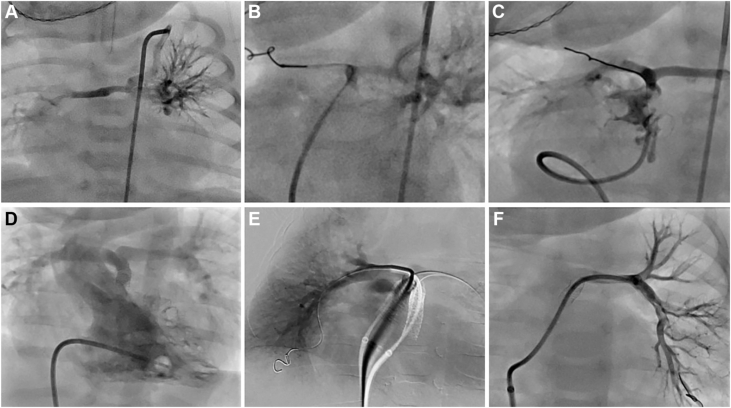
**Case 3.** Serial angiograms demonstrate the progression of pulmonary artery (PA) growth following right ventricular outflow tract stenting. (**A**-**C**) Initial catheterization at 3 weeks of age. (**A**) Angiogram showing diminutive native PA opacified via a systemic-to-pulmonary collateral arising from the left subclavian artery. (**B**) A BMW wire is shown traversing the pulmonary valve and twisted/locked into the right upper PA to facilitate intervention. (**C**) Poststent angiogram showing immediate improvement in the caliber of the true PA with a Nakata index of 23.5 (**D**-**F**). Follow-up catheterization at 3 months of age. (**D**) Right ventricular angiogram showing marked improvement in the size of the PA, though a focal stenosis is noted distal to the right ventricular outflow tract stent. (**E**, **F**) Selective angiograms of the right PA and the left PA after crossing the stent, revealing significant improvement in branch PA caliber with a Nakata index of 105.46. Note the substantial increase in vessel size between the initial catheterization (**A**) and the 3-month follow-up (**D**), reflecting effective recruitment and growth of the native PA.

A male infant was diagnosed prenatally with PA/VSD/MAPCA. A CT scan performed shortly after birth revealed multiple MAPCA supplying both the right and left lungs, with a Nakata index of 23.5. Because of good oxygen saturation at 86%, intervention was delayed for a few weeks, and the baby was discharged home and brought to the catheterization lab for an elective procedure at 3 weeks of age (weight 3.75 kg). An angiogram was performed through a MAPCA from the left subclavian artery, and another simultaneous angiogram was performed in the RVOT using an angled glide catheter to determine the proximity of the MPA and the RVOT. The distance between the RVOT stump and the MPA was 0.6 mm. A Renegade micro catheter was placed through the angled glide catheter in the RVOT through which a BMW wire was passed across the atretic pulmonary valve into the distal right upper PA and twisted/locked in the lung parenchyma. Serial balloon angioplasty was performed with a 1.5 mm × 20 mm and 3 mm × 20 mm NC emerge, followed by successful deployment of a 4 mm × 15 mm Onyx Frontier stent across the RVOT and pulmonary valve. The final angiography confirmed a patent RVOT with antegrade flows into both PA (Figure 8A-C).

At 3 months of age, the patient (weight 7.3 kg) underwent repeat catheterization. Angiograms showed significant branch PA growth with improved arborization of the branch PA (RPA 4.9 mm, LPA 4.3 mm; Nakata index 105.46 with a discrete narrowing beneath the stent). A 5.5 GuideLiner (Vascular Solution Inc) with the aid of a 3.5 mm balloon was used to cross the original stent and facilitate placement of a 5 mm × 18 mm Onyx Frontier stent (final diameter 5.3 mm). After placement, the degree of overlap between the 2 stents was not satisfactory. To ensure stability and secure the more proximal stent, an additional stent (5 mm × 22 mm Onyx Frontier) was deployed (Figure 8D-F).

At 5 months of age, he underwent cardiac catheterization (RPA 6.7 mm and LPA 6.5 mm; Nakata index 146). At 8 months of age, he underwent complete repair; however, at this time, no follow-up CT or catheterization is available to report on the new Nakata index after repair (Supplemental Video 3).

## Case 4 (unsuccessful case)

**Figure 9:**
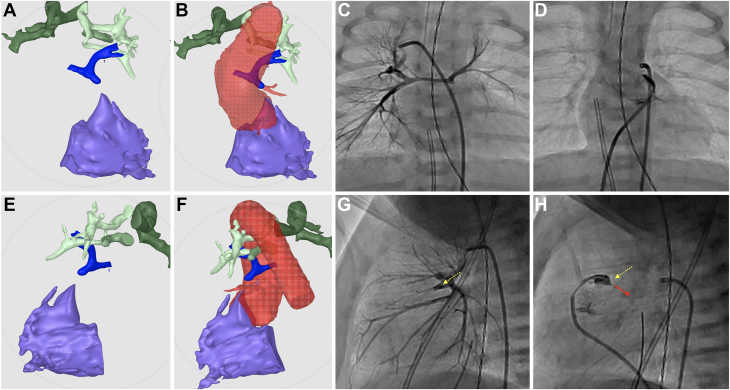
**Case 4, an unsuccessful case.** Panels (**A**-**D**): anteroposterior (AP) projections and corresponding 3D representations. The 3D models (**A**, **B**) show the pulmonary arteries in blue, the right ventricle in purple, the aorta in red, and the aortopulmonary collaterals in green. Note the separation between the right ventricular outflow tract (RVOT) stump and the true pulmonary artery (PA), as well as the close proximity of a coronary artery branch in this projection. Angiographic images (**C**, **D**) correlate these findings with (**C**) showing filling of the hypoplastic PA via a collateral injection and (**D**) showing the small RVOT stump. Note how the main pulmonary artery (MPA) stump points leftward and the RVOT stump points rightward. Panels (**E**-**H**): lateral projections and corresponding 3D representations. The 3D models (**E**, **F**) highlight the spatial gap between the true PA and the RVOT stump, as well as the nearby coronary artery course. The lateral angiograms (**G**, **H**) show the misalignment between the RVOT stump and the true MPA stump. The red arrow denotes the RVOT stump trajectory. The RVOT stump points more posterior, inferior, and rightward, while the yellow dotted arrow denotes the MPA stump trajectory pointing more anterior, superior, and leftward, illustrating the malalignment between the 2 structures.

A female infant diagnosed prenatally with PA/VSD/MAPCA was taken to the catheterization lab at 2 weeks of age (weight 3.1 kg). During the catheterization, it was noted that the “beak-like” infundibular RVOT and the take-off of the MPA appeared malaligned, and the distance between the RVOT stump and the MPA was 3.2 mm. Crossing was attempted using a BMW wire and a Renegade microcatheter, as in our prior cases, but was unsuccessful. Given the distance and malalignment between the RVOT and MPA, as well as the proximity of a coronary artery branch, radiofrequency perforation was not pursued, and the patient was referred for a Blalock-Thomas-Taussig shunt (Figure 9).

## Discussion

The management of PAHP and MAPCA remains a complex and evolving field, with ongoing debate regarding the optimal surgical and interventional strategies to achieve a low-resistance pulmonary vascular bed and long-term survival.^17^^,^^18^ This case series of 4 patients with PAHP, 3 with PA/VSD and MAPCA, and 1 with PA/IVS, highlights innovative strategies to establish pulmonary blood flow and promote PA growth. These cases underscore the importance of early, tailored interventions to prepare the pulmonary vascular bed for definitive repair, aligning with recent trends favoring a multistage approach combining catheter-based and surgical techniques.^17^^,^^19^^,^^20^ Equally important, careful patient selection—based on anatomic suitability for RVOT stenting, MAPCA-to-PA connection dilation, and subsequent surgical augmentation—is critical to achieving successful outcomes.

Traditionally, the repair involves surgical unifocalization of MAPCA to integrate collateral vessels into the native PA system.^7^^,^^9^^,^^18^^,^^20^ One prominent example is the Stanford group’s midline unifocalization strategy, which has been applied to a large cohort with excellent outcomes. Their approach prioritizes early, single-stage repair through surgical unifocalization of MAPCA, resulting in high rates of complete repair and satisfactory right ventricular pressures.^21^ However, this method reflects a highly individualized, center-specific practice that relies on specialized expertise and infrastructure. As such, although it demonstrates impressive innovation, it is not easily standardized or broadly reproducible.

In contrast, the group in Melbourne advocates a fundamentally different philosophy: that true PA are almost always present—even if severely hypoplastic—and should be aggressively sought. Their strategy relies on early central end-to-side aortopulmonary shunting to promote native PA growth, minimizing the need for unifocalization.^5^^,^^22^ This philosophy resonates with this case series, which supports a shift toward native PA rehabilitation through the restoration of antegrade, pulsatile flow—even in patients with MAPCA.

Additionally, there is increasing recognition that RVOT stenting can be particularly effective in stimulating native PA growth in the setting of tetralogy of Fallot, where it may outperform traditional surgical shunts.^6^^,^^9^^,^^14^^,^^19^

Our experience with this approach indicates that both proximity and alignment of the MPA and RVOT are critical determinants of feasibility. The distance between the RVOT and MPA should be carefully evaluated on both CT and simultaneous angiography, as CT imaging may overestimate the gap. CT-fluoroscopy fusion can serve as an alternative when the distal MPA stump cannot be entered, though CT alignment with live fluoroscopy may be cumbersome. Systems enabling 3D rotational angiography during the procedure may provide more accurate and user-friendly guidance.^23^

In our experience, a coaxial system with a soft BMW wire can be safely attempted even at longer distances; however, radiofrequency perforation should be reserved for cases in which the 2 structures are within millimeters and well aligned to reduce the risk of extravascular injury. In our series, the RVOT-MPA stump distance was <1 mm in all successful cases, supporting this approach.

Notably, in 1 patient, balloon dilation of a MAPCA-to-native PA connection enabled perfusion of that lung segment through the native vasculature without the need for surgical unifocalization. To the best of our knowledge, this is the first report of such a technique, marking a novel step in the management of complex PA/MAPCA anatomy. However, the approach is only possible when there is a very small connection between a MAPCA and the native PA. This connection is too small to be considered a true dual supply. Dilating the connection brings that lung territory into the central PA circulation—territory that would otherwise be lost if the MAPCA were not unifocalized. In contrast, when a territory is supplied only by a MAPCA with no connection, surgical unifocalization is required. Patient selection, therefore, depends on finding and crossing these tiny connections, which should be carefully looked for at each catheterization, as these connections can grow with time.

In this case series, a particularly striking case involved a unique scenario^24^ of PA/IVS, hypoplastic PA, and tiny MAPCA in which this technique resulted in an increase in Nakata index from 13.38 to 227, reflecting significant PA growth and highlighting the potential of early catheter-based interventions to rehabilitate the pulmonary vascular bed in extreme cases. To the best of our knowledge, this combination of anatomy and recovery trajectory has not been previously reported.

Pulmonary artery enlargement in this cohort occurred in a stepwise and complementary fashion. RVOT opening and stenting served as the critical first step, providing the initial stimulus for growth and converting severely hypoplastic vessels into arteries of adequate caliber for subsequent reconstruction. In selected patients, balloon dilation of MAPCA-PA connections further expanded the pulmonary vascular bed by incorporating territories that would otherwise have been excluded, thereby enhancing overall growth potential. Definitive repair with surgical plasty, often supplemented by additional catheter-based balloon dilations, then produced further enlargement and optimization of the PA (Central Illustration). This sequence illustrates how catheter-based strategies can establish the foundation on which surgical interventions build, with RVOT stenting and MAPCA-PA connection dilation creating the conditions necessary for later surgical augmentation and long-term success.

**Central Illustration:**
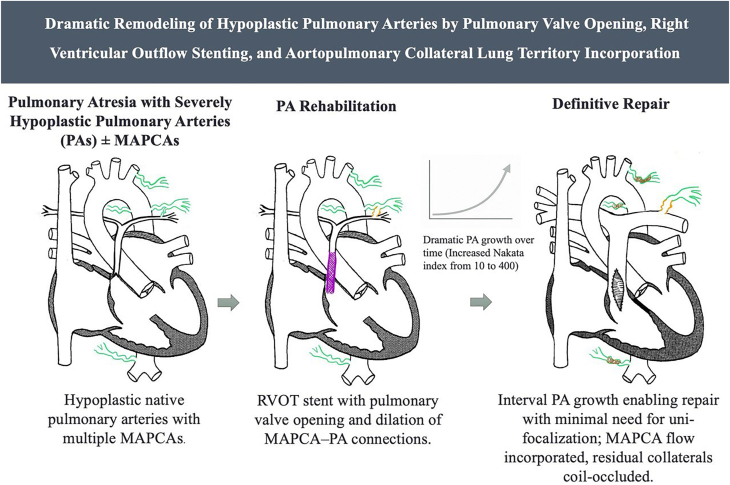
Progressive pulmonary artery (PA) rehabilitation in PA ± MAPCA, showing right ventricular outflow tract (RVOT) stenting, major aortopulmonary collateral arteries (MAPCA)-PA dilation, and subsequent PA growth enabling definitive repair (Nakata index increase from 10 to 400).

Balloon dilation of small MAPCA-PA communications carries important risks, as MAPCA have abnormal vascular architecture and may be prone to limited expansion, dissection, or rupture. In this case, extremely gradual, serial balloon dilation was performed to minimize the risk of injury. This technique should be undertaken with caution, as distal vessel injury may be difficult to control—particularly in segments where covered stents cannot be deployed, and coil occlusion of the involved lung territory may ultimately be required.

Our findings emphasize the evolving paradigm in PAHP management, favoring early, staged interventions tailored to individual anatomy. The novel use of balloon dilation to incorporate MAPCA into the native PA system and the successful treatment of a rare PA/IVS phenotype demonstrate the potential of innovative catheter-based techniques to improve outcomes. These cases advocate for multidisciplinary collaboration and careful risk-benefit assessment to expand therapeutic options, particularly in anatomically complex patients.

## Conclusion

Establishing prograde flow using pulmonary valve opening and RVOT stenting, followed by serial balloon dilation of segmental branches, including dilation of interpulmonary to collateral connections when present, can result in dramatic growth and improved arborization of the PA. This strategy may enhance surgical repair and, in some cases, negate the need for unifocalization.

## Supplementary material

Supplemental Video 1 Case 1: Illustrated summary of Case 1, a 6-year-old patient with PA/IVS, a hypoplastic RV, diminutive native PAs, and tiny MAPCAs. The video traces her serial catheterizations, beginning with the initial procedure at 8 days of age, which shows retrograde filling of severely hypoplastic PAs via a right lower lobe pulmonary vein wedge angiogram. It then demonstrates successful crossing and stenting of the pulmonary valve following simultaneous angiograms on both sides of the valve. Subsequent interventions are shown, including serial balloon dilations and PA rehabilitation up to her first surgery at 6 months of age, when an RVOT patch was placed. Her final catheterization at 3 years of age, following PA plasty and bioprosthetic valve placement, demonstrates markedly improved PA anatomy. Notably, the Nakata index improved over time from 13.38 (day of life 8) to 84 (preoperative), and 227 (latest follow-up), reflecting substantial PA growth with staged intervention.

Supplemental Video 2 Case 2: Illustrated summary of Case 2, a 14-month-old with PA/VSD, and MAPCAs. The video presents serial catheterizations, beginning with an aortogram demonstrating a large MAPCA supplying both lungs. A tiny connection between the MAPCA and the true PAs is identified (highlighted with a red arrow). A microcatheter is advanced across this connection to selectively opacify the native PAs; its course from the MAPCA into the true PAs is indicated by blue dotted and yellow striped arrows. Simultaneous angiograms on both sides of the pulmonary valve are performed, followed by successful valve crossing using a BMW wire. The wire is twisted/locked distally in the right lower PA to facilitate RVOT stenting. At the second catheterization, performed at 3 months of age, the connection between the true LPA and the large MAPCA is balloon-dilated, effectively incorporating the MAPCA into the native pulmonary circulation and promoting PA recruitment and growth.

Supplemental Video 3 Case 3: Illustrated summary of Case 3, a 3-month-old with PA/VSD, and MAPCAs. It shows engagement of the left subclavian collateral, followed by dual angiography via the RVOT and MAPCA, revealing hypoplastic native PAs. A BMW wire traverses the pulmonary valve using a locking technique, allowing advancement of a Renegade catheter into the RPA. Balloon dilation and RVOT stenting are performed without a long sheath. At 3-month follow-up, branch PA growth is demonstrated, and a second stent is deployed using the GuideLiner technique across the prior RVOT stent.

## References

[1] Ikai A. (2018). Surgical strategies for pulmonary atresia with ventricular septal defect associated with major aortopulmonary collateral arteries. Gen Thorac Cardiovasc Surg.

[2] Fouilloux V., Bonello B., Kammache I., Fraisse A., Macé L., Kreitmann B. (2012). Management of patients with pulmonary atresia, ventricular septal defect, hypoplastic pulmonary arteries and major aorto-pulmonary collaterals: focus on the strategy of rehabilitation of the native pulmonary arteries. Arch Cardiovasc Dis.

[3] Stumper O., Ramchandani B., Noonan P. (2013). Stenting of the right ventricular outflow tract. Heart.

[4] Metras D., Chetaille P., Kreitmann B., Fraisse A., Ghez O., Riberi A. (2001). Pulmonary atresia with ventricular septal defect, extremely hypoplastic pulmonary arteries, major aorto–pulmonary collaterals. Eur J Cardiothorac Surg.

[5] d'Udekem Y. (2019). Rehabilitation of pulmonary arteries in pulmonary atresia, VSD and MAPCAs. Oper Tech Thorac Cardiovasc Surg.

[6] Quandt D., Ramchandani B., Penford G. (2017). Right ventricular outflow tract stent versus BT shunt palliation in tetralogy of Fallot. Heart.

[7] Tchervenkov C.I., Salasidis G., Cecere R. (1997). One-stage midline unifocalization and complete repair in infancy versus multiple-stage unifocalization followed by repair for complex heart disease with major aortopulmonary collaterals. J Thorac Cardiovasc Surg.

[8] Petrucci O., O'Brien S.M., Jacobs M.L., Jacobs J.P., Manning P.B., Eghtesady P. (2011). Risk factors for mortality and morbidity after the neonatal Blalock-Taussig shunt procedure. Ann Thorac Surg.

[9] Prakoso R., Citra Dewi R., Mendel B. (2024). Clinical outcomes of right ventricular outflow tract stenting compared to surgical shunting in late-presenting children. Front Cardiovasc Med.

[10] Dohlen G., Chaturvedi R.R., Benson L.N. (2009). Stenting of the right ventricular outflow tract in the symptomatic infant with tetralogy of Fallot. Heart.

[11] Dreger N.J., Herrick N.L., Guyon P.W., Moore J.W., El-Said H.G. (2022). Pulmonary atresia with intact ventricular septum: from radiofrequency perforation to transcatheter pulmonary valve. J Invasive Cardiol.

[12] Ovaert C., Qureshi S.A., Rosenthal E., Baker E.J., Tynan M. (1998). Growth of the right ventricle after successful transcatheter pulmonary valvotomy in neonates and infants with pulmonary atresia and intact ventricular septum. J Thorac Cardiovasc Surg.

[13] Walsh M.A., Lee K.J., Chaturvedi R., Van Arsdell G.S., Benson L.N. (2007). Radiofrequency perforation of the right ventricular outflow tract as a palliative strategy for pulmonary atresia with ventricular septal defect. Catheter Cardiovasc Interv.

[14] Aurigemma D., Moore J.W., Vaughn G., Moiduddin N., El-Said H.G. (2018). Perforation and right ventricular outflow tract stenting: alternative palliation for infants with pulmonary atresia/ventricular septal defect. Congenit Heart Dis.

[15] Abbaszadeh R., Askari-Moghadam R., Moradian M. (2023). The Nakata index and McGoon ratio: correlation with the severity of pulmonary regurgitation after the repair of paediatric tetralogy of Fallot. Egypt Heart J.

[16] Hesari M., Ng’eno M., Gordon B.M. (Published online May 5, 2025). The wire twisting/locking technique to facilitate precise PDA stent delivery in neonates with ductal-dependent pulmonary blood flow. Pediatr Cardiol.

[17] De Giovanni J.V. (2004). Timing, frequency, and results of catheter intervention following recruitment of major aortopulmonary collaterals in patients with pulmonary atresia and ventricular septal defect. J Interv Cardiol.

[18] Malhotra S.P., Hanley F.L. (2009). Surgical management of pulmonary atresia with ventricular septal defect and major aortopulmonary collaterals: a protocol-based approach. Semin Thorac Cardiovasc Surg Pediatr Card Surg Annu.

[19] Barwad P., Prasad K., Santosh K., Bootla D., R P.C., Naganur S.H. (2020). Showing the right path to the flow is possible. Pulmonary valve perforation with RVOT stenting in a sick infant. IHJ Cardiovasc Case Rep (CVCR).

[20] Sandoval J.P., Chaturvedi R.R., Benson L. (2016). Right ventricular outflow tract stenting in tetralogy of Fallot infants with risk factors for early primary repair. Circ Cardiovasc Interv.

[21] Mainwaring R.D., Patrick W.L., Hanley F.L. (2019). Surgical management of pulmonary atresia with ventricular septal defect and major aortopulmonary collateral arteries: part I—anatomy, physiology, and palliative procedures. Oper Tech Thorac Cardiovasc Surg.

[22] Watterson K.G., Wilkinson J.L., Karl T.R., Mee R.B. (1991). Very small pulmonary arteries: central end-to-side shunt. Ann Thorac Surg.

[23] Sivakumar K., Mumtaz Z.A., Sagar P. (2022). Application of Vessel Navigator™ fusion imaging software in a complex transcatheter palliation of tetralogy of Fallot with pulmonary atresia. Ann Pediatr Cardiol.

[24] Luciani G.B., Swilley S., Starnes V.A. (1995). Pulmonary atresia, intact ventricular septum, and major aortopulmonary collaterals: morphogenetic and surgical implications. J Thorac Cardiovasc Surg.

